# Interpretable SVM-Based Integrated Ultrasound Model for Preoperative Thyroid Nodule Subtype Classification: Improved Identification of Follicular Variant Papillary Thyroid Carcinoma

**DOI:** 10.3390/diagnostics16131950

**Published:** 2026-06-23

**Authors:** Ran Zheng, Zhen Wang, Yongxin Li, Yuanqing Zhang, Fang Nie

**Affiliations:** 1 Department of Ultrasound, Lanzhou University Second Hospital, Lanzhou 730030, China; zr642002872@163.com (R.Z.); wz17794203660@163.com (Z.W.); yuanqinzh@163.com (Y.Z.); 2 Gansu Province Clinical Research Center for Ultrasonography, Lanzhou 730030, China; 3 Gansu Province Medical Engineering Research Center for Intelligence Ultrasound, Lanzhou 730030, China; 4 School of Automation and Intelligence, Beijing Jiaotong University, Beijing 100044, China; liyongxin951120@163.com; 5 Department of Thoracic Surgery, Beijing Genertec Aerospace Hospital, Beijing 100074, China

**Keywords:** ultrasound, radiomics, follicular variant papillary thyroid carcinoma, classical papillary thyroid carcinoma, benign thyroid nodules

## Abstract

**Background/Objectives:** Preoperative differentiation among benign thyroid nodules, follicular variant papillary thyroid carcinoma (FV-PTC), and classical papillary thyroid carcinoma (C-PTC) remains clinically challenging. FV-PTC is particularly difficult to identify due to its substantial sonographic and cytological overlap with both benign nodules and other malignant subtypes, frequently resulting in overtreatment or delayed diagnosis. This study aimed to develop and validate an interpretable multimodal model for accurate three-class discrimination using routine ultrasound images, with a specific focus on improving the preoperative identification of FV-PTC. **Methods:** This retrospective study included 479 pathologically confirmed thyroid nodules from 462 patients. Conventional ultrasound features and radiomics features extracted from grayscale ultrasound and color Doppler flow imaging were used to construct three predictive models: a Conventional Ultrasound model (conventional ultrasound features only), a Radiomics model (radiomics features only), and an Integrated model (combined features). Each model was trained using four machine learning classifiers. Model performance was evaluated using the area under the receiver operating characteristic curve (AUC), accuracy, sensitivity, specificity, positive predictive value (PPV), negative predictive value (NPV), and F1 score. Model interpretability was assessed using SHapley Additive exPlanations (SHAP) analysis, and clinical usefulness was evaluated using decision curve analysis (DCA). **Results:** The support vector machine (SVM)-based Integrated Model achieved the best overall performance. In the independent testing cohort, the AUCs were 0.853 for FV-PTC, 0.882 for C-PTC and 0.928 for benign nodules. The Integrated Model showed the greatest improvement for FV-PTC, with a ΔAUC of 0.141 compared with the Conventional Ultrasound Model. SHAP (SHapley Additive exPlanations) analysis identified wavelet-HL_gldm_Dependence and wavelet-HH_glcm_InverseVariance as the two most important radiomics predictors in both the Radiomics Model and the Integrated Model, demonstrating robust cross-model stability and high discriminative power. **Conclusions:** The SVM-based Integrated Model demonstrated promising performance for three-class classification of thyroid nodules and enhanced the preoperative identification of FV-PTC. This approach may provide an interpretable and noninvasive decision-support tool for refining subtype-specific risk stratification and supporting individualized clinical management.

## 1. Introduction

Thyroid nodules are among the most common endocrine disorders encountered in clinical practice, identified in up to 50–60% of the general population with the widespread use of high-resolution ultrasonography [1,2]. Accurate preoperative characterization of thyroid nodules is clinically important, as benign nodules are usually managed conservatively, whereas malignant nodules often require surgery and risk-adapted postoperative treatment [3]. Beyond distinguishing benign from malignant nodules, histological subtyping of PTC is increasingly relevant for individualized risk assessment, as emphasized by the 2022 World Health Organization (WHO) classification [4]. C-PTC (55–75%) and FV-PTC (12–30%) are the most prevalent PTC subtypes, representing the majority of diagnosed cases [2]. C-PTC is more commonly associated with lymphatic invasion and locoregional lymph node metastasis. In contrast, FV-PTC is generally indolent and more prone to hematogenous dissemination, with occasional cases showing aggressive progression and distant metastasis [5,6]. Consistently, a statistically significantly lower percentage of persistent disease was found in the FV-PTC than in the C-PTC (*p* < 0.0001) [5]. Differences in radioactive iodine (RAI) uptake have also been observed [7]. Therefore, accurate preoperative differentiation among benign nodules, C-PTC and FV-PTC is of substantial importance for optimizing patient management.

**Figure 1:**
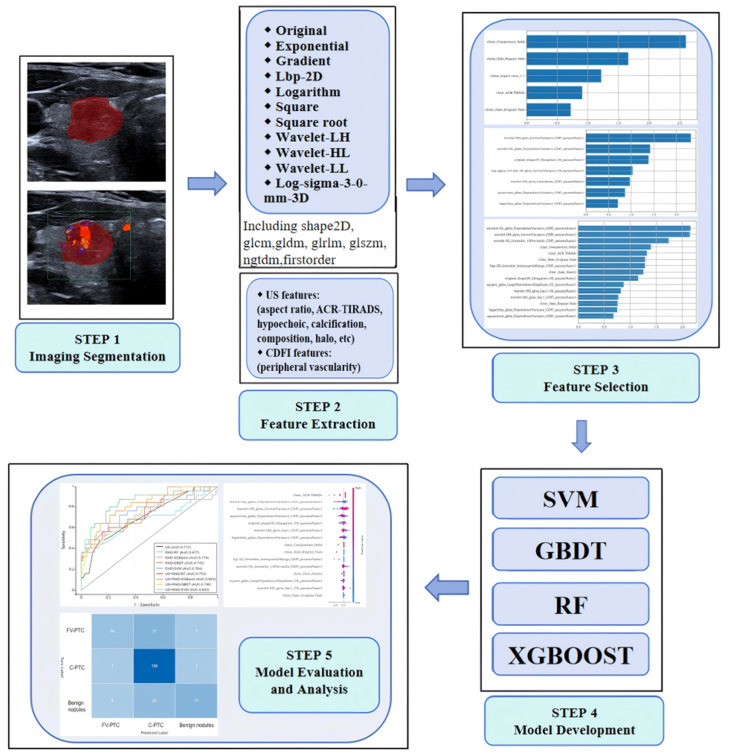
Workflow of the models. SVM, Support Vector Machine; RF, Random Forest; GBDT, Gradient Boosting Decision Trees; XGBoost, eXtreme Gradient Boosting.

Despite its clinical importance, reliable preoperative differentiation among these entities remains challenging [8]. FV-PTC represents a particular diagnostic challenge and is more likely to be missed or misclassified preoperatively. Although fine-needle aspiration biopsy (FNAB) is effective for diagnosing C-PTC, it frequently yields indeterminate results for FV-PTC due to cytomorphological overlap with benign follicular lesions [9]. In addition, molecular testing is of limited assistance, as FV-PTC exhibits a lower prevalence of BRAF V600E mutations compared with C-PTC [10,11]. Ultrasonography remains the first-line imaging modality for evaluating thyroid nodules because of its accessibility, noninvasiveness, and real-time imaging capability [12]. Moreover, FV-PTC often lacks the classic sonographic features associated with malignancy, resulting in substantial imaging overlap with both benign follicular-patterned lesions and C-PTC. Radiomics enables high-throughput extraction of quantitative image features beyond visual assessment and may complement conventional ultrasound evaluation [13]. Recent artificial intelligence and radiomics studies have shown promising performance in thyroid nodule diagnosis [14]. However, few studies have addressed the more clinically challenging three-way differentiation among benign nodules, FV-PTC, and C-PTC [15]. Furthermore, limited model interpretability remains a critical barrier to clinical application. Therefore, this study aimed to develop and validate an interpretable integrated model for the preoperative classification of these thyroid nodule subtypes (Figure 1), with particular emphasis on the diagnostically challenging FV-PTC.

## 2. Materials and Methods

### 2.1. Patients

This retrospective study was conducted in accordance with the Declaration of Helsinki and was approved by the Ethics Committee of Lanzhou University Second Hospital. The requirement for informed consent was waived because this study used retrospectively collected anonymized data.

We retrospectively reviewed patients with thyroid nodules who underwent preoperative ultrasound and subsequent thyroid surgery at Lanzhou University Second Hospital between March 2020 and March 2025. During this period, all eligible FV-PTC and benign nodules were consecutively enrolled, while eligible C-PTC nodules were randomly sampled from the same source population using a computer-generated randomization procedure. A total of 462 patients with 479 thyroid nodules were included, including 127 FV-PTC, 217 C-PTC, and 135 benign nodules. The dataset was randomly divided at the patient level into a training cohort and an internal testing cohort at a 4:1 (approximately 80:20) ratio, resulting in 383 and 96 nodules, respectively. This split was chosen to provide a sufficiently large training set for high-dimensional radiomics feature selection and classifier optimization while maintaining an independent internal testing cohort, particularly given the limited number of FV-PTC cases. The training set included 383 nodules, consisting of 102 FV-PTC, 173 C-PTC, and 108 benign thyroid nodules. The validation set included 96 nodules, consisting of 25 FV-PTC, 44 C-PTC, and 27 benign thyroid nodules. All nodules from the same patient were allocated to the same cohort to avoid data leakage.

**Figure 2:**
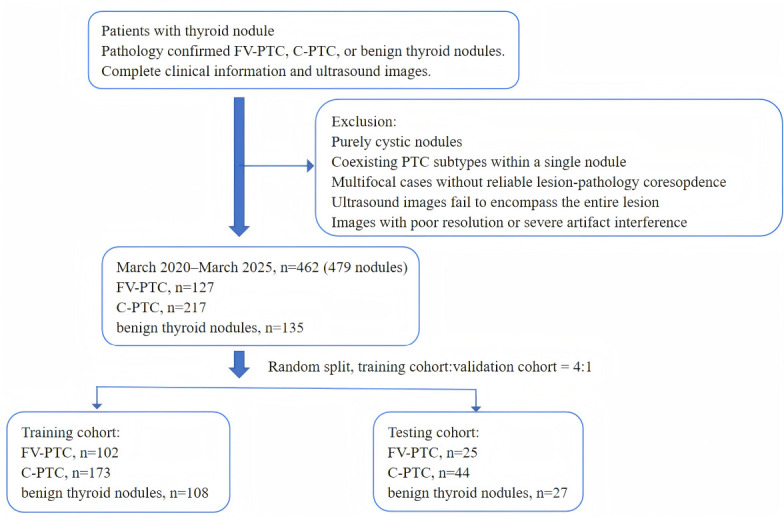
Flow diagram of the study population. PTC, papillary thyroid carcinoma.

The inclusion criteria were: (1) patients who underwent thyroid surgery with postoperative pathological confirmation and (2) complete clinical information and preoperative ultrasound images. The exclusion criteria were: (1) purely cystic nodules; (2) coexistence of different PTC subtypes within a single nodule; (3) multifocal disease without reliable lesion-pathology matching; (4) incomplete lesion coverage on ultrasound images; and (5) poor-quality images or severe artifacts. The dataset was randomly divided at the patient level into a training cohort and an internal testing cohort at an 8:2 ratio, resulting in 383 and 96 nodules, respectively. The training cohort was used exclusively for model development and feature selection, whereas the internal testing cohort was used for independent performance evaluation. All nodules from the same patient were allocated to the same cohort. The study flowchart is shown in Figure 2.

### 2.2. Pathological Diagnosis

The final diagnosis of all included thyroid nodules was confirmed by postoperative histopathological examination. Because preoperative fine-needle aspiration biopsy data were not consistently available in this retrospective cohort, they were not included in the analysis. Pathological diagnosis followed the 2022 WHO Classification. FV-PTC included both infiltrative and encapsulated subtypes, whereas non-invasive follicular thyroid neoplasm with papillary-like nuclear features (NIFTP) was excluded.

### 2.3. Thyroid Ultrasound Examination

All ultrasound examinations were performed by radiologists with more than 5 years of experience in thyroid ultrasound. Grayscale ultrasound and color Doppler flow imaging (CDFI) were acquired using either an iU22 scanner (Philips Medical Systems, Bothell, WA, USA) equipped with a 5–12 MHz linear probe or an ACUSON Sequoia scanner (Siemens Healthineers, Malvern, PA, USA) with a 4–10 MHz linear probe. The acquired images were archived for subsequent analysis.

### 2.4. Nodule Segmentation and Conventional Ultrasound Feature Assessment

Regions of interest (ROIs) for all thyroid nodules were manually delineated on both grayscale ultrasound and CDFI images using the Darwin Research Platform. Two radiologists with more than 5 years of experience in thyroid ultrasound, who were blinded to the pathological diagnoses, independently delineated the regions of interest and assessed the conventional ultrasound features. Any discrepancies were discussed, and necessary modifications were made to achieve consensus. Conventional ultrasound features were assessed on grayscale ultrasound and CDFI images by the same two radiologists. The evaluated grayscale ultrasound features included margin, aspect ratio, halo, hypoechogenicity, composition, and microcalcifications. American College of Radiology Thyroid Imaging Reporting and Data System (ACR TI-RADS) was also included as a candidate predictor because of its established clinical relevance in thyroid nodule risk stratification. The CDFI feature evaluated was peripheral vascularity.

### 2.5. Conventional Ultrasound Feature Selection and Model Development

Conventional sonographic features were imported into this platform (https://arxiv.org/abs/2009.00908, accessed on 27 July 2025) for analysis in the training cohort only. A linear SVM with L1 regularization (one-vs-rest strategy for multiclass classification) was used in the training cohort to select conventional ultrasound features and construct the Conventional Ultrasound Model. All candidate conventional ultrasound features were entered into the model, and the optimal regularization parameter C was selected by 5-fold cross-validation in the training cohort. L1 regularization was employed to reduce the impact of potential multicollinearity among correlated variables. Features with non-zero coefficients were retained to build the Conventional Ultrasound Feature SVM Model. The model was then evaluated in the testing cohort.

### 2.6. Radiomics Feature Extraction and Selection

After manual segmentation of each thyroid nodule, radiomics features were extracted from grayscale ultrasound (B-mode) and CDFI images using the PyRadiomics package in PyRadiomics version 3.0.1 in Python (Python Software Foundation, Beaverton, OR, USA). The extracted radiomics features included Shape2D features, first-order features, and second-order texture features, with the latter including features derived from the gray-level dependence matrix (GLDM), gray-level co-occurrence matrix (GLCM), gray-level size zone matrix (GLSZM), gray-level run-length matrix (GLRLM), and neighboring gray-tone difference matrix (NGTDM). Eight image filters, including logarithm, square, square root, wavelet, exponential, gradient, local binary pattern (LBP), and Laplacian of Gaussian (LoG), were applied to generate transformed images for radiomics feature extraction. We extracted a total of 1125 radiomics features from each ROI, including 18 shape 2D features, 432 first-order features and 675 second-order features. Because both B-mode ultrasound and CDFI images were analyzed for each nodule, 2250 radiomics features were obtained for each nodule in total. To evaluate feature reproducibility, a randomly selected subset of 50 nodules from the training cohort was independently segmented by two radiologists, and intraclass correlation coefficients (ICCs) were calculated for the extracted features. Only features with ICC > 0.75 were retained for subsequent analysis. All radiomics features were normalized using min-max scaling based on the training cohort, and the same scaling parameters were subsequently applied to the testing cohort. An analysis of variance (ANOVA) F-test was performed exclusively in the training cohort to filter features based on statistical significance, with a significance cutoff (*p*-value) of 0.05. Subsequently, stability selection based on 100 bootstrap resamples was performed to identify robust radiomics features, and only features selected in more than 50% of resamples were retained for further analysis. All preprocessing steps were strictly performed using the training cohort only.

### 2.7. Radiomics Model and Integrated Model Development and Evaluation

Two modeling strategies were developed for thyroid nodule classification. The first was a radiomics model based exclusively on selected radiomics features, and the second was an integrated model combining radiomics features with conventional ultrasound features. For each modeling strategy, four machine-learning classifiers, including Support Vector Machine (SVM), Random Forest (RF), Gradient Boosting Decision Trees (GBDT), and eXtreme Gradient Boosting (XGBoost), were trained and compared to identify the optimal classifier. To improve generalizability and mitigate overfitting, nonlinear kernels were used in SVM, and the maximum tree depth was restricted to five for all tree-based models (RF, GBDT, and XGBoost). Model performance was comprehensively evaluated using the AUC, sensitivity, specificity, positive predictive value (PPV), negative predictive value (NPV), F1 score, and accuracy (ACC). AUCs were calculated using a one-vs-rest (OVR) strategy for each subtype. To further enhance model interpretability, SHapley Additive exPlanations (SHAP) were used to quantify feature contributions and interpret the predictions of the best-performing model. The DeLong test was used to compare the AUCs between different models in an OVR manner for each subtype. Additional pairwise ROC analyses were performed among FV-PTC, C-PTC, and benign nodules. For each comparison, only the two corresponding subgroups were included. The AUC, 95% CI, *p* value, sensitivity, and specificity were calculated for each pairwise comparison. DCA was performed to evaluate the clinical net benefit. The outputs of all classifiers were converted into class probabilities.

### 2.8. Statistical Analysis

All statistical analyses were performed using SPSS software (version 27.0; IBM Corp., Armonk, NY, USA). The normality of continuous variables was assessed before analysis. Continuous variables are presented as mean ± standard deviation (SD) or median (interquartile range), depending on their distribution. Categorical variables are presented as number (%). Comparisons among the three pathological groups were performed using one-way ANOVA for normally distributed continuous variables or the Kruskal–Wallis test for non-normally distributed variables. Categorical variables were compared using the chi-square test or Fisher’s exact test. In particular, the distributions of categorical ultrasound features across the FV-PTC, C-PTC, and benign nodule groups were compared using Pearson’s chi-square test. All statistical tests were two-sided, and *p* < 0.05 was considered statistically significant. Radiomics analyses were performed using Python (version 3.6).

## 3. Result

### 3.1. Baseline Characteristics of the Three Pathological Groups

A total of 479 thyroid nodules from 462 patients were included in this study, comprising 127 FV-PTC, 217 C-PTC, and 135 benign nodules. The baseline clinical characteristics are summarized in Table 1. The dataset was divided into a training cohort (*n* = 383) and a testing cohort (*n* = 96). The overall mean age was 48.4 ± 10.8 years, and 74.1% (355/479) of nodules were female. Patients with benign nodules were significantly older than those with FV-PTC and C-PTC (53.5 ± 10.8 vs. 48.4 ± 9.5 vs. 45.4 ± 11.4 years, *p* < 0.001). No significant differences were observed in nodule location among the three pathological subtypes (*p* > 0.05). The baseline characteristics and conventional ultrasound features of the training and testing cohorts are summarized in Table 2. No statistically significant differences were observed between the two cohorts in demographic characteristics or conventional ultrasound features (all *p* > 0.05), suggesting that the two cohorts were generally comparable.

### 3.2. The Prediction Performance of the Conventional Ultrasound Model

Table 3 illustrates the ultrasound features of the included thyroid nodule subtypes. In the training cohort, multiple ultrasound features demonstrated significant differences among the three pathological subtypes, including margin, aspect ratio, echogenicity, composition, halo pattern, ACR TI-RADS, and peripheral vascularity (all *p* < 0.05). The Conventional Ultrasound Model included ACR TI-RADS, halo, aspect ratio and composition. In the testing cohort, the Conventional Ultrasound Model achieved OVR AUCs of 0.712 (95% CI: 0.598–0.825), 0.788 (95% CI: 0.702–0.874), and 0.861 (95% CI: 0.778–0.944) for FV-PTC, C-PTC, and benign nodules, respectively, as shown in Table 4. The corresponding comparisons were FV-PTC versus non-FV-PTC nodules, C-PTC versus non-C-PTC nodules, and benign nodules versus non-benign nodules. Among the three classification tasks, the model showed the lowest discriminative performance for FV-PTC compared to C-PTC and benign nodules.

### 3.3. The Prediction Performance of the Radiomics Model and the Integrated Model

**Figure 3:**
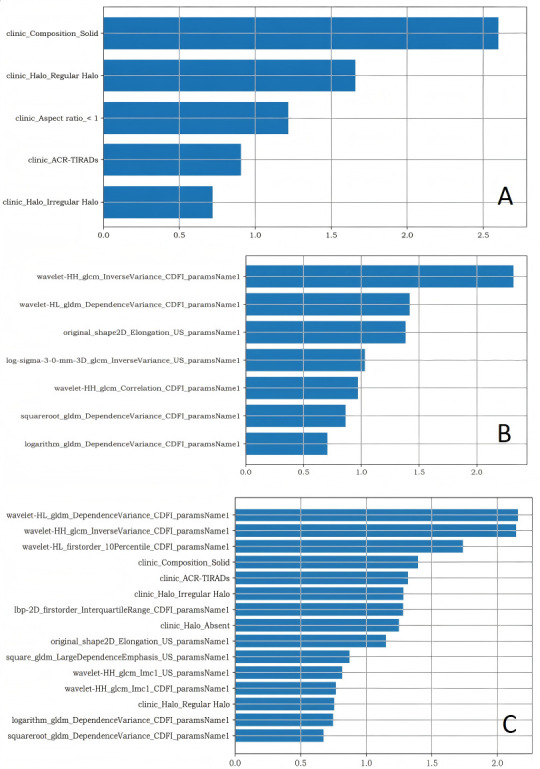
Feature importance of the Conventional Ultrasound Model (**A**), the Radiomics Model (**B**) and the Integrated Model (**C**). Feature-importance analysis based on SelectFromModel identified wavelet-HL_gldm_Dependence and wavelet-HH_glcm_InverseVariance as two consistently important radiomics predictors in both the Radiomics Model and the Integrated Model. GLDM, gray-level dependence matrix; GLCM, gray-level co-occurrence matrix.

SHAP analysis identified wavelet-HL_gldm_Dependence and wavelet-HH_glcm_InverseVariance as two consistently important radiomics predictors in both the Radiomics Model and the Integrated Model (Figure 3). Figure 3 shows the feature importance rankings for the Conventional Ultrasound Model (A), the Radiomics Model (B) and the Integrated Model (C). The *x*-axis represents the dimensionless coefficient/importance score output by SelectFromMode. Larger values indicate greater relative feature contribution to subtype classification. GLDM and GLCM texture patterns consistently ranked as the top two contributors across models.

The diagnostic performances of the Radiomics Model and Integrated Model in the testing cohort are summarized in Table 4. The detailed performance metrics of the SVM-based Integrated Model in both the training and testing cohorts are summarized in Table 5. Overall, the Integrated Model achieved higher class-specific AUCs than the Conventional Ultrasound Model and generally outperformed the Radiomics Model in the testing cohort. Considering the overall balance of AUC, F1 score, and accuracy across the three classes, the SVM-based Integrated Model was selected as the final model. In the testing cohort, the SVM-based Integrated Model, which combined selected radiomics features with conventional ultrasound features, achieved OVR AUCs of 0.853 (95% CI: 0.758–0.948) for differentiating FV-PTC from non-FV-PTC nodules, 0.882 (95% CI: 0.816–0.947) for differentiating C-PTC from non-C-PTC nodules, and 0.928 (95% CI: 0.861–0.995) for differentiating benign nodules from malignant PTC subtypes, as shown in Table 4. In the testing cohort, pairwise ROC analysis showed significant discrimination across all subgroup comparisons. The AUCs were 0.805 for FV-PTC versus C-PTC, 0.892 for FV-PTC versus benign nodules, and 0.937 for C-PTC versus benign nodules. All *p* values were <0.001 (Table 6).

### 3.4. Performance Comparison of the Conventional Ultrasound, Radiomics, and Integrated Models

**Figure 4:**
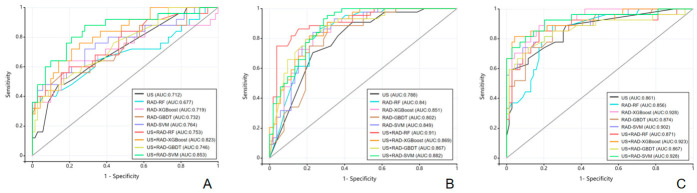
ROC curves of the constructed models in the testing cohort. ROC, receiver operating characteristic; (**A**): FV-PTC; (**B**): C-PTC; (**C**): Benign nodules.

**Figure 5:**
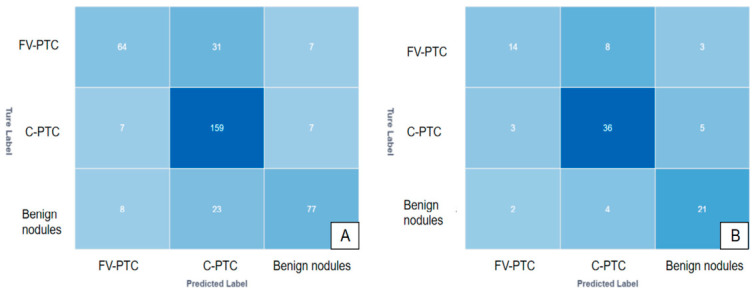
Confusion matrices of the SVM-based Integrated Model in the training cohort (**A**) and internal testing cohort (**B**).

ROC curve analysis showed that the Integrated Model generally provided better discriminative performance than the Conventional Ultrasound Model and the Radiomics Model in the testing cohort (Figure 4). For FV-PTC, both the Conventional Ultrasound Model and the Radiomics Model showed limited performance, whereas the Integrated Model achieved improved classification capability. For benign nodules, all models demonstrated relatively high diagnostic performance, with AUC values exceeding 0.85. The confusion matrices (Figure 5) provide additional insight into the classification performance of the Integrated Model across subtypes.

**Figure 6:**
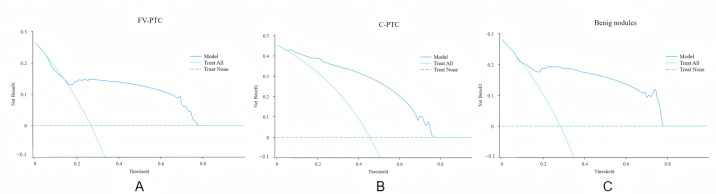
The DCA of the SVM-based Integrated model (blue line). The “treat-all” and “treat-none” strategies are shown as reference lines. (**A**): FV-PTC; (**B**): C-PTC; (**C**): Benign nodules.

**Figure 7:**
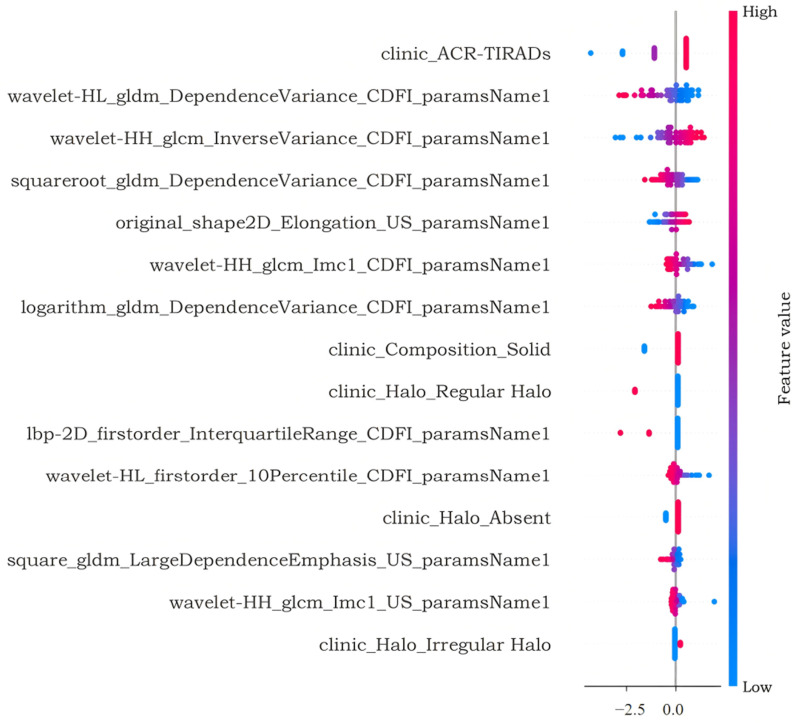
SHAP summary plot demonstrating the contribution of features to the final SVM-based Integrated Model. Features are ranked in descending order according to their mean absolute SHAP values, with higher-ranked features indicating greater overall contributions to model prediction. Each dot represents an individual case, and the horizontal position corresponds to the SHAP value, reflecting the direction and magnitude of the feature’s effect on the model output.

Pairwise comparisons using the DeLong test further confirmed that the Integrated Model significantly outperformed the Conventional Ultrasound Model across all subtypes. Compared with the Conventional Ultrasound Model, the Integrated Model showed the largest improvement in FV-PTC (ΔAUC = 0.141), followed by C-PTC (ΔAUC = 0.098) and benign nodules (ΔAUC = 0.067) in the testing cohort (Table 7). DCA showed that the Integrated Model consistently provided greater net benefit than the “treat-all” and “treat-none” strategies. This benefit was observed across clinically relevant threshold probabilities. For FV-PTC and benign nodules, the threshold range was approximately 0.2–0.8 (Figure 6A,C). For C-PTC, the range was 0.1–0.7 (Figure 6B). The most stable performance was observed for C-PTC. These results suggest that the Integrated Model offers superior clinical utility across all pathological subtypes, enabling more effective risk stratification than either universal intervention or non-intervention. SHAP analysis demonstrated that ACR TI-RADS was the most influential predictor in the SVM-based integrated model, suggesting that conventional ultrasound risk stratification remained a major contributor to model prediction. Several radiomic texture features, including wavelet-HL_gldm_DependenceVariance and wavelet-HH_glcm_InverseVariance, also ranked highly, indicating that quantitative image-derived features related to intranodular heterogeneity and gray-level distribution provided additional predictive information (Figure 7). Overall, these findings indicate that integrating radiomics features with conventional ultrasound features improves multiclass classification performance, particularly for the diagnostically challenging FV-PTC subtype.

## 4. Discussion

In this study, we developed and validated the SVM-based Integrated Model for the noninvasive preoperative classification of thyroid nodules. In the independent testing cohort, the SVM-based Integrated Model achieved OVR AUCs of 0.928 for distinguishing benign from non-benign nodules, 0.853 for distinguishing FV-PTC from non-FV-PTC nodules, and 0.882 for distinguishing C-PTC from non-C-PTC nodules, as shown in Table 5. DeLong analysis showed that the Integrated Model significantly improved the discrimination of FV-PTC from non-FV-PTC nodules compared with the Conventional Ultrasound Model (ΔAUC = 0.141). This finding highlights the potential value of the Integrated Model for the preoperative identification of this diagnostically challenging subtype.

Compared with previous studies, our study addresses thyroid nodule diagnosis in a more clinically representative setting by simultaneously evaluating benign nodules, C-PTC, and FV-PTC. Prior studies reported high AUC values of 0.85–0.96 for discriminating benign from malignant thyroid nodules, but subtype-specific information remained limited [16,17]. Several studies have further explored subtype-specific diagnosis. Ng et al. developed an ultrasound-based risk scoring system to differentiate C-PTC from FV-PTC, calculated as 5 × hypoechogenicity + 3 × calcification + 3 × irregular margin. This score achieved an AUC of 0.85 for predicting C-PTC, with a specificity of 87% and a sensitivity of 69% at the optimal cut-off value of 8.0 [18]. Hou et al. combined deep learning with surface-enhanced Raman spectroscopy (SERS) analysis of FNAB samples and achieved high accuracy in PTC subtype classification [19]. In addition, Shen et al. leveraged state-of-the-art deep learning methods to classify follicular-patterned neoplasms, grouping FV-PTC with follicular carcinoma as malignant. The model showed strong diagnostic performance, with an AUC of 0.853 in the validation set [20]. Building on previous studies, our study simultaneously evaluates multiple pathological subtypes, thereby more closely reflecting the diagnostic complexity encountered in routine clinical practice.

The SVM-based Integrated Model showed the most significant diagnostic improvement for FV-PTC, with a ΔAUC of 0.141 compared with the Conventional Ultrasound Model. The observed improvement is clinically meaningful, given the well-recognized difficulty of preoperatively identifying FV-PTC. Several studies have reported subtype-related sonographic features in PTC, but substantial overlap among subtypes limits the reliability of conventional ultrasound for definitive subtype classification [21,22]. This limitation is particularly evident for FV-PTC, which often resembles benign thyroid nodules and lacks typical suspicious features such as microcalcifications, marked hypoechogenicity, and taller-than-wide shape [22]. Consistent with these findings, our conventional ultrasound model showed only moderate performance for FV-PTC, with an AUC of 0.712 [9,23,24]. FNAB and molecular testing are also limited for FV-PTC identification. C-PTC and FV-PTC share the hallmark nuclear features of PTC. However, C-PTC is typically characterized by true papillary architecture, cellular swirls, and psammoma bodies. In contrast, FV-PTC predominantly exhibits a follicular growth pattern. Psammoma bodies are usually absent in FV-PTC, and nuclear atypia tends to be milder and more focally distributed [4,25]. Consequently, FV-PTC may show substantial cytological overlap with benign follicular-patterned lesions on FNAB [26]. Molecular testing can provide complementary diagnostic information, but it is insufficient as a standalone approach for reliable FV-PTC identification. C-PTC is strongly associated with BRAF V600E mutations, whereas FV-PTC is more commonly driven by RAS-family alterations [27]. This molecular distinction underscores the limitation of a BRAF-only diagnostic approach, which may miss FV-PTC and other RAS-driven tumors. For example, in Bethesda III thyroid nodules, BRAF V600E was detected in only 40% of FV-PTC cases, compared with nearly 90% of C-PTC cases [28].

The improved performance of the SVM-based Integrated Model may be explained by the complementary value of conventional ultrasound features and radiomics features [29]. Radiomics enables the extraction of high-dimensional features that reflect lesion heterogeneity, which may correspond to underlying histopathological complexity [30]. In the present study, SHAP analysis identified wavelet-HL_gldm_Dependence and wavelet-HH_glcm_InverseVariance as two important radiomics predictors in both the Radiomics Model and the Integrated Model, suggesting stable cross-model discriminative value. The former feature may reflect aspects of lesion texture homogeneity, whereas the latter may capture certain patterns of gray-level distribution. ACR TI-RADS also showed subtype-related discriminatory value. Similar to the findings reported by Chen et al., approximately 45–50% of FV-PTC cases in our cohort were classified as ACR TI-RADS 5, whereas the remaining cases were categorized as ACR TI-RADS 3 or 4. In contrast, approximately 85–90% of C-PTC nodules were assigned to ACR TI-RADS 5 [24]. This distribution suggests that C-PTC more frequently presents with highly suspicious ultrasound features captured by ACR TI-RADS. Consistent with these findings, Scappaticcio et al. demonstrated significant differences in ACR TI-RADS categorization between classic and non-classic PTC. However, this subtype-discriminatory value was primarily observed in nodules measuring ≥10 mm [31]. Therefore, ACR TI-RADS may provide valuable subtype-related information and complement quantitative radiomics features in an integrated diagnostic model [32,33].

SHAP analysis further enhanced model interpretability by quantifying the contribution of individual predictors to the final model output. DCA further suggested that the Integrated Model may offer clinical benefit across a range of threshold probabilities. By improving preoperative subtype classification, the proposed model may complement existing ultrasound-based risk stratification systems and support more individualized clinical decision-making. Because it relies on routinely acquired ultrasound images, the model has potential for integration into preoperative imaging workflows.

There are a few limitations to the current study. First, the surgical cohort provided a reliable standard for pathological subtype classification. However, it may introduce selection bias, as surgically treated nodules are typically more suspicious. This may limit generalizability and potentially overestimate diagnostic performance. Therefore, the findings should be interpreted in clinically selected cases. Second, the SVM-based Integrated Model showed significant pairwise discrimination among FV-PTC, C-PTC, and benign nodules in the test cohort, supporting its potential as an auxiliary tool for subtype differentiation. However, external validation is still needed, particularly given the relatively lower discrimination between FV-PTC and C-PTC. Third, rarer thyroid tumor variants were not included and should be evaluated in future studies.

## Figures and Tables

**Table 1:** Baseline clinical characteristics of the three pathological groups.

Variables	FV-PTC (*n* = 127)	C-PTC (*n* = 217)	Benign Nodules (*n* = 135)	*p* Value
Gender, *n* (%)				
Male	41 (32.3)	61 (28.1)	22 (16.3)	0.008
Female	86 (67.7)	156 (71.9)	113 (83.7)	
Age, mean ± SD (years)	48.38 ± 9.53	45.37 ± 11.39	53.47 ± 10.84	<0.001
Location, *n* (%)				0.165
Left lobe	48 (37.8)	89 (41)	65 (48.1)	
Right lobe	67 (52.8)	111 (51.2)	66 (48.9)	
Isthmus	12 (9.4)	17 (7.8)	4 (3)	

**Table 2:** Comparison of baseline characteristics and conventional ultrasound features between the training and testing cohorts.

Variables	Training Set (*n* = 383)	Testing Set (*n* = 96)	*p* Value
Age, mean ± SD (years)	48.16 ± 11.53	49.63 ± 10.08	0.214
Gender, *n* (%)			0.629
Male	101 (26.4)	23 (24.0)	
Female	282 (73.6)	73 (76.0)	
Location, *n* (%)			0.161
Left lobe	154 (40.2)	48 (50.0)	
Right lobe	200 (52.2)	44 (45.8)	
Isthmus	29 (7.6)	4 (4.2)	
Margin, *n* (%)			0.198
regular	95 (24.8)	30 (31.2)	
irregular	288 (75.2)	66 (68.8)	
Aspect ratio, *n* (%)			0.630
<1	162 (42.3)	38 (39.6)	
≥1	221 (57.7)	58 (60.4)	
Microcalcifications, *n* (%)			0.155
Yes	149 (38.9)	45 (46.9)	
No	234 (61.1)	51 (53.1)	
Hypoechoic, *n* (%)			0.451
Yes	255 (66.6)	60 (62.5)	
No	128 (33.4)	36 (37.5)	
Composition, *n* (%)			0.671
Solid	341 (89.0)	84 (87.5)	
Cystic-solid	42 (11.0)	12 (12.5)	
Halo, *n* (%)			0.579
Irregular Halo	66 (17.2)	17 (17.7)	
Regular Halo	18 (4.7)	7 (7.3)	
Absent	299 (78.1)	72 (75.0)	
ACR-TIRADS, *n* (%)			0.549
2	6 (1.6)	1 (1.0)	
3	13 (3.4)	6 (6.3)	
4	99 (25.8)	27 (28.1)	
5	265 (69.2)	62 (64.6)	
Peripheral vascularity, *n* (%)			0.051
Yes	53 (13.8)	21 (21.9)	
No	330 (86.2)	75 (78.1)	

**Table 3:** Conventional ultrasound features of the three pathological groups in the training and testing cohorts. * Pearson’s chi-square test; º Fisher’s exact test. *p* values were calculated using the appropriate statistical test.

Variables	FV-PTC (*n* = 102)	C-PTC (*n* = 173)	Benign Nodules (*n* = 108)	*p* Value	FV-PTC (*n* = 25)	C-PTC (*n* = 44)	Benign Nodules (*n* = 27)	*p* Value
	**Training cohort (** ***n*** **= 383)**				**Testing cohort (** ***n*** **= 96)**			
Margin, *n* (%)				<0.0001 *				0.0003 *
regular	24 (23.5)	19 (11.0)	52 (48.1)		8 (32.0)	6 (13.6)	16 (59.3)	
irregular	78 (76.5)	154 (89.0)	56 (51.9)		17 (68.0)	38 (86.4)	11 (40.7)	
Aspect ratio, *n* (%)				<0.0001 *				<0.0001 *
<1	45 (44.1)	44 (25.4)	73 (67.6)		9 (36.0)	8 (18.2)	21 (77.8)	
≥1	57 (55.9)	129 (74.6)	35 (32.4)		16 (64.0)	36 (81.8)	6 (22.2)	
Microcalcifications, *n* (%)				<0.0001 *				0.0004 *
Yes	27 (26.5)	95 (54.9)	27 (25.0)		9 (36.0)	30 (68.2)	6 (22.2)	
No	75 (73.5)	78 (45.1)	81 (75.0)		16 (64.0)	14 (31.8)	21 (77.8)	
Hypoechoic, *n* (%)				<0.0001 *				<0.0001 *
Yes	74 (72.5)	152 (87.9)	29 (26.9)		18 (72.0)	38 (86.4)	4 (14.8)	
No	28 (27.5)	21 (12.1)	79 (73.1)		7 (28.0)	6 (13.6)	23 (85.2)	
Composition, *n* (%)				<0.0001 *				<0.0001 º
Solid	100 (98.0)	172 (99.4)	69 (63.9)		24 (96.0)	43 (97.7)	17 (63.0)	
Cystic-solid	2 (2.0)	1 (0.6)	39 (36.1)		1 (4.0)	1 (2.3)	10 (37.0)	
Halo, *n* (%)				<0.0001 º				<0.0001 º
Irregular Halo	31 (30.4)	21 (12.1)	14 (13.0)		10 (40.0)	2 (4.5)	5 (18.5)	
Regular Halo	1 (1.0)	0 (0)	17 (15.7)		0 (0)	0 (0)	7 (25.9)	
Absent	70 (68.6)	152 (87.9)	77 (71.3)		15 (60.0)	42 (95.5)	15 (55.6)	
ACR-TIRADS, *n* (%)				<0.0001 º				<0.0001 º
2	0 (0)	0 (0)	6 (5.6)		0 (0)	0 (0)	1 (3.7)	
3	3 (2.9)	0 (0)	10 (9.3)		0 (0)	0 (0)	6 (22.2)	
4	33 (32.4)	17 (9.8)	49 (45.4)		10 (40.0)	7 (15.9)	10 (37.0)	
5	66 (64.7)	156 (90.2)	43 (39.8)		15 (60.0)	37 (84.1)	10 (37.0)	
Peripheral vascularity, *n* (%)				<0.0001 *				<0.0001 *
Yes	10 (9.8)	10 (5.8)	33 (30.6)		2 (8.0)	5 (11.4)	14 (51.9)	
No	92 (90.2)	163 (94.2)	75 (69.4)		23 (92.0)	39 (88.6)	13 (48.1)	

Percentages may not total 100.0% due to rounding.

**Table 4:** One-vs-rest AUCs of the constructed models in the testing cohort. SVM, Support Vector Machine; RF, Random Forest; GBDT, Gradient Boosting Decision Trees; XGBoost, eXtreme Gradient Boosting.

Model	FV-PTC	C-PTC	Benign Nodules
Integrated Model			
SVM AUC (95% CI)	0.853 (0.758, 0.948)	0.882 (0.816, 0.947)	0.928 (0.861, 0.995)
GBDT AUC (95% CI)	0.746 (0.633, 0.859)	0.867 (0.795, 0.939)	0.867 (0.770, 0.963)
RF AUC (95% CI)	0.753 (0.635, 0.871)	0.910 (0.850, 0.970)	0.871 (0.777, 0.964)
XGBoost AUC (95% CI)	0.823 (0.730, 0.915)	0.869 (0.800, 0.939)	0.923 (0.854, 0.991)
Radiomics Model			
SVM AUC (95% CI)	0.764 (0.644, 0.884)	0.849 (0.771, 0.927)	0.902 (0.832, 0.973)
GBDT AUC (95% CI)	0.732 (0.607, 0.858)	0.802 (0.713, 0.892)	0.874 (0.795, 0.954)
RF AUC (95% CI)	0.677 (0.537, 0.818)	0.840 (0.760, 0.920)	0.856 (0.776, 0.935)
XGBoost AUC (95% CI)	0.719 (0.582, 0.856)	0.851 (0.775, 0.927)	0.928 (0.876, 0.980)
Conventional Ultrasound Model			
SVM AUC (95% CI)	0.712 (0.598, 0.825)	0.788 (0.702, 0.874)	0.861 (0.778, 0.944)

**Table 5:** Performance metrics of the SVM-based Integrated Model for predicting different thyroid nodule subtypes in the training and testing cohorts. AUCs were calculated using a one-vs-rest strategy. PPV, positive predictive value; NPV, negative predictive value; ACC, accuracy.

	AUC (95% CI)	Sensitivity	Specificity	PPV	NPV	ACC	F1
Training set							
FV-PTC	0.846 (0.796, 0.895)	0.627	0.947	0.810	0.875	0.862	0.707
C-PTC	0.883 (0.850, 0.917)	0.919	0.743	0.746	0.918	0.822	0.824
Benign nodules	0.885 (0.842, 0.928)	0.713	0.949	0.846	0.894	0.883	0.774
Testing set							
FV-PTC	0.853 (0.758, 0.948)	0.560	0.930	0.737	0.857	0.833	0.636
C-PTC	0.882 (0.816, 0.947)	0.818	0.769	0.750	0.833	0.792	0.783
Benign nodules	0.928 (0.861, 0.995)	0.778	0.884	0.724	0.910	0.854	0.750

**Table 6:** Pairwise ROC analysis of the SVM-fusion model for differentiating FV-PTC, C-PTC, and benign nodules in the testing cohort.

Subtypes	*n*	AUC (95% CI)	*p* Value	Sensitivity	Specificity
FV-PTC vs. C-PTC	25 vs. 44	0.805 (0.679–0.930)	<0.001	0.720	0.841
FV-PTC vs. Benign nodules	25 vs. 27	0.892 (0.799–0.985)	<0.001	0.875	0.815
C-PTC vs. Benign nodules	44 vs. 27	0.937 (0.871–1.000)	<0.001	0.886	0.889

**Table 7:** DeLong test results comparing the diagnostic performance between the Integrated Model and the Conventional Ultrasound Model. AUCs were calculated using a one-vs-rest strategy.

Subtype	AUC Difference	Std. Error	95% CI	z	*p* Value
FV-PTC	0.1414	0.0643	0.017–0.269	2.2305	0.0257
C-PTC	0.0979	0.0434	0.013–0.183	2.2566	0.0240
Benign nodules	0.0666	0.0477	−0.027–0.160	1.3942	0.1633

## Data Availability

The data that support the findings of this study are available from the corresponding author upon reasonable request. The data are not publicly available due to patient privacy protection.

## Abbreviations

The following abbreviations are used in this manuscript:

PTC	papillary thyroid carcinoma
C-PTC	classical papillary thyroid carcinoma
FV-PTC	follicular variant papillary thyroid carcinoma
FNAB	fine-needle aspiration biopsy
BRAF	B-Raf proto-oncogene, serine/threonine kinase
RAS	rat sarcoma viral oncogene homolog
CDFI	color Doppler flow imaging
B-mode	brightness-mode ultrasound
ACR TI-RADS	American College of Radiology Thyroid Imaging Reporting and Data System
SVM	support vector machine
RF	random forest
GBDT	gradient boosting decision trees
XGBoost	extreme gradient boosting
SHAP	SHapley Additive exPlanations
GLDM	gray-level dependence matrix
GLCM	gray-level co-occurrence matrix
WHO	World Health Organization
